# Pre-eruptive Coronal Resorptions as a Clinical Feature of *FAM83H*-Related Amelogenesis Imperfecta: Insights from Two Brazilian Families

**DOI:** 10.1007/s00223-026-01540-8

**Published:** 2026-05-04

**Authors:** Kemelly Karolliny Resende, Luanna de Sousa Amorim, Lilian Marly de Paula, André Ferreira Leite, Juliana Forte Mazzeu, Paulo Marcio Yamaguti, Ana Carolina Acevedo

**Affiliations:** 1 Department of Dentistry, Laboratory of Oral Histopathology, Faculty of Health Sciences, University of Brasilia, Brasilia, Brazil; 2 Oral Care Center for Inherited Diseases, University Hospital of Brasilia, Brasilia, Brazil; 3 Department of Dentistry, Division of Radiology, Faculty of Health Sciences, University of Brasilia, Brasilia, Brazil; 4 Laboratory of Clinical Genetics, Faculty of Medicine, University of Brasília, Brasília, Brazil

**Keywords:** Amelogenesis Imperfecta, FAM83H, Tooth impaction, Pre-eruptive crown resorption, Whole-exome sequencing

## Abstract

Amelogenesis Imperfecta (AI) is a group of rare hereditary conditions characterized by quantitative and/or qualitative enamel defects affecting both primary and permanent dentitions. Among the more than 70 genes associated with AI, *FAM83H* is the only gene known to cause autosomal dominant hypocalcified AI (ADHCAI). Recent studies have shown that causative variants in *FAM83H* disrupt amelogenesis and may also affect dental follicle cells, potentially leading to tooth impaction in ADHCAI patients. Here, we report two unrelated Brazilian patients with ADHCAI who present a distinctive and severe phenotype characterized by delayed eruption, multiple impacted teeth, and pre-eruptive crown resorptions (PECR). Longitudinal radiographic analysis revealed multiple unerupted teeth with progressive PECR. Using whole-exome sequencing, we identified two nonsense heterozygous *FAM83H* causative variants (c.1055 C > A, p.Ser352*; c.1379G > A, p.Trp460*). Our findings represent the first report of *FAM83H*-related ADHCAI in Brazilian families and expand both the phenotypic spectrum and clinical severity associated with this gene. The presence of widespread PECR and adjacent bone alterations suggests that *FAM83H* dysfunction may affect not only enamel formation but also tooth eruption pathways and local tooth–bone interactions. This study highlights the importance of early diagnosis, individualized radiographic follow-up, and genetic testing to guide counselling and clinical management of affected individuals.

**Supplementary Information:**

The online version contains supplementary material available at 10.1007/s00223-026-01540-8.

## Introduction

Amelogenesis Imperfecta (AI) encompasses a diverse group of rare hereditary conditions characterized by developmental defects in dental enamel formation, manifesting as qualitative, quantitative, or combined anomalies in both primary and permanent dentition. AI can occur as an isolated condition or can be associated with syndromic presentations involving systemic alterations, with varied inheritance patterns including X-linked, autosomal dominant, and autosomal recessive modes [1]. Clinically, AI is typified by features such as reduced enamel thickness, a rough surface texture, enamel pits or grooves, and mineralization defects including hypomineralization or hypocalcification. Beyond these primary enamel defects, patients may also present with additional findings, including delayed tooth eruption, prolonged retention of primary teeth, anterior open bite, gingival fibromatosis, impacted teeth, and pre-eruptive crown resorption (PECR) [2].

The molecular etiology of AI is heterogeneous, with over 70 genes associated with both isolated and syndromic forms [3]. Notably, autosomal dominant hypocalcified Amelogenesis Imperfecta (ADHCAI), also referred to as AI type IIIa according to Witkop’s classification (OMIM #130900), is uniquely attributed to causative variants in the *FAM83H* gene (OMIM #611927). The *FAM83H* gene (8q24.3) comprises five exons and encodes a 1,179–amino acid protein, whose translation begins in exon 2, with exon 5 contributing most of the coding sequence (933 amino acids). As in other members of the FAM83 family, the N-terminal region contains a conserved Domain of Unknown Function 1669 (DUF1669; amino acids 17–285), whereas the extensive C-terminal region (amino acids 286–1179) is intrinsically disordered but functionally essential for enamel formation [4, 5]. All causative *FAM83H* variants reported to date in ADHCAI occur exclusively in exon 5, particularly within the segment encoding Ser287–Glu694. Most of these variants are nonsense or frameshift mutations that introduce premature termination codons (PTCs), generating truncated proteins that lack the critical C-terminal domain [6]. Because these PTCs arise in the final exon, the resulting transcripts typically escape nonsense-mediated mRNA decay (NMD) [7], enabling the production of stable truncated proteins. The persistence of these aberrant protein products results in a dominant gain-of-function effect, which disrupts normal ameloblast function and constitutes the central pathogenic mechanism underlying *FAM83H-related* AI [8–11].

Distinguishing it from many other AI-related genes that encode extracellular matrix proteins, *FAM83H* encodes a non-secreted, intracellular protein that is typically localized in the cytoplasm and frequently associated with perinuclear vesicles or the Golgi apparatus [12]. The truncated FAM83H proteins aberrantly lose their physiological cytoplasmic localization and instead translocate to the nucleus, a mechanism that is hypothesized to disrupt the normal enamel mineralization process [13]. The clinical presentation of ADHCAI resulting from *FAM83H* variants is characterized by enamel of normal thickness that is severely weakened, highly susceptible to wear, and exhibits a rough, brown or yellow‑brown discoloration [14]. Beyond the primary enamel defects, affected individuals may also present variable craniofacial phenotypes, such as an anterior open bite, although this feature is not consistently observed in all affected families. Further dental anomalies, including tooth impaction and pre-eruptive crown resorption (PECR), have been documented in individuals carrying *FAM83H* variants [14, 15]. Consequently, this study sought to delineate the clinical and molecular findings in two unrelated Brazilian patients presenting with ADHCAI, severe tooth impaction, and PECR.

## Materials and Methods

This study was performed in compliance with the Declaration of Helsinki and was approved by the Ethics Committee of the Faculty of Health Sciences, University of Brasília, Brazil. All participants and, when applicable, their parents signed informed consent for participation in the study, including authorization for molecular analysis and for photographs documenting their clinical presentation. Patients underwent physical, oral, and radiographic examinations, and intraoral photographs were obtained. Blood samples were collected, and genomic DNA was extracted from peripheral blood lymphocytes using the salting-out method, and DNA samples of the proband patients were subjected to exome sequencing [16]. The exome sequencing was performed at Genesis Genomics (Brazil), and variant analysis was conducted using the Franklin platform (Genoox ^®^). All variants were classified according to the latest guidelines of the American College of Medical Genetics (ACMG) [17]. The nomenclature of all variants was verified using the Mutalyzer website (https://mutalyzer.nl*).* Teeth were classified as impacted if they failed to erupt within the expected chronological sequence. The diagnosis of PECR and enlarged dental follicle was performed via Cone-Beam Computed Tomography (CBCT) since CBCT allows for the visualization of details not observed in conventional radiographs.

.

## Results

### Family 1

**Fig. 1:**
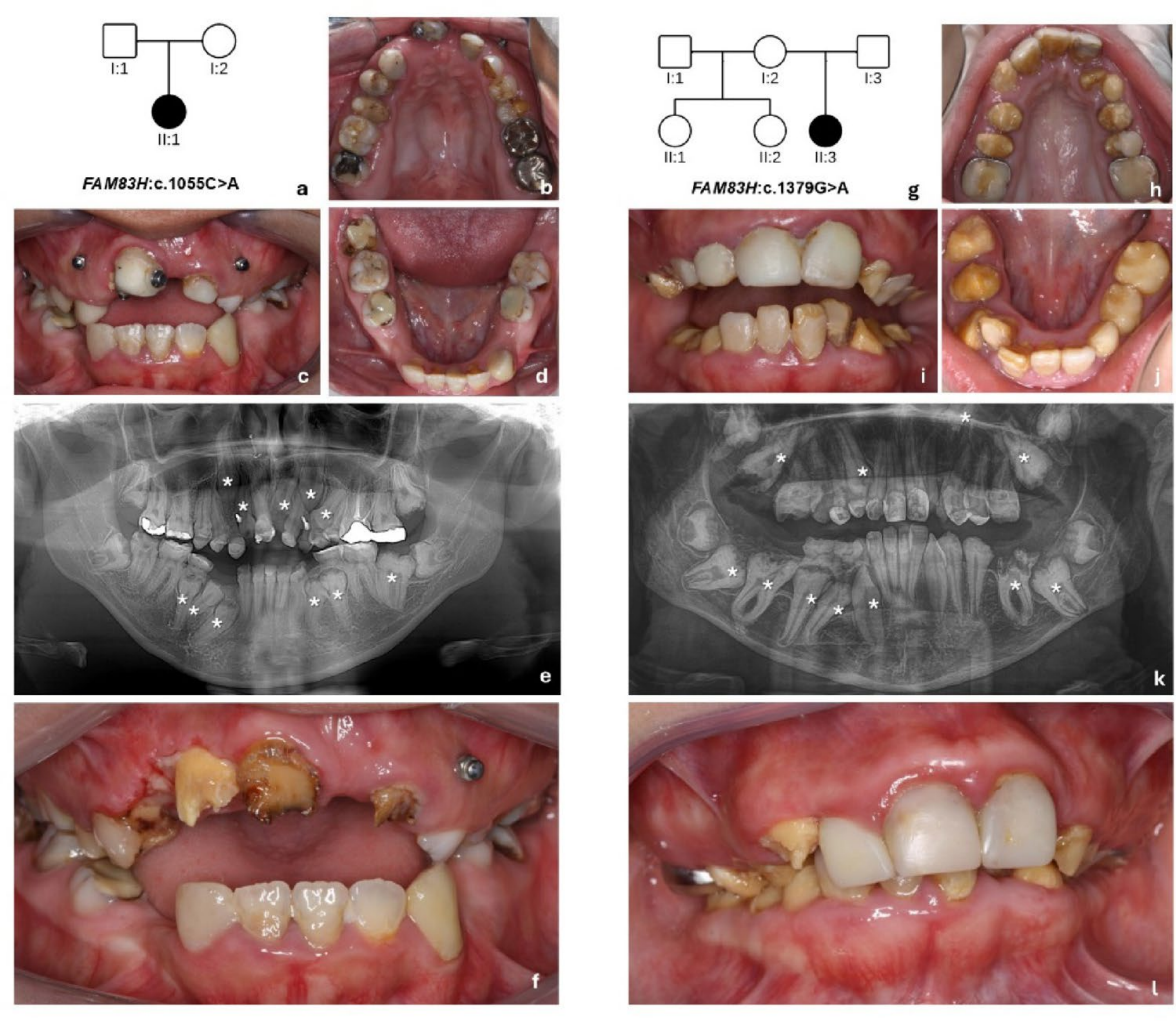
**a** Family pedigree of Patient 1 (II:1). **b**–**d** Intraoral photographs of Patient 1 at age 18, showing anterior open bite and clinical features of hypocalcified AI. Incisors had been restored with composite resin, and the patient presented some mini-implants due to a previous but unsuccessful orthodontic treatment. **e** In the panoramic radiograph, eleven impacted teeth were detected (*). **f** After gingivectomy in the region of tooth 12 and removal of the composite resin, it was possible to observe the rough enamel surface of the maxillary incisors due to PECR. **g** Family pedigree of patient 2 (II:3). **h**–**j** Intraoral photographs at age 15 showing the anterior open bite and hypocalcified AI. **k** Observe the disturbed eruption pattern in the maxilla and mandible, and the presence of eleven impacted teeth (*). **l** In a recently erupted canine, the crown surface presented an irregular shape with grooves probably due to PECR

The proband was an 18-year-old Brazilian female (II:1), born to non-consanguineous unaffected parents (Fig. 1a), referred to the Oral Care Center for Inherited Diseases at the University Hospital of Brasília, Brazil, due to delayed tooth eruption and suspected AI. She had previous medical diagnoses of attention-deficit/hyperactivity disorder (ADHD) and asthma.

Intraoral and extraoral examination at age 18 revealed facial dysmorphia, anterior open bite, prolonged retention of primary teeth, and delayed eruption of permanent teeth (Fig. 1a–f). Teeth showed yellow-brown discoloration, surface irregularities, and altered morphology, consistent with a hypocalcified AI phenotype (Fig. 1f). Many teeth had undergone previous restorative treatment, limiting the evaluation of their real morphology. Orthodontic mini-implants had been installed to extrude the impacted teeth without success, which were removed (Fig. 1c and e).

**Fig. 2:**
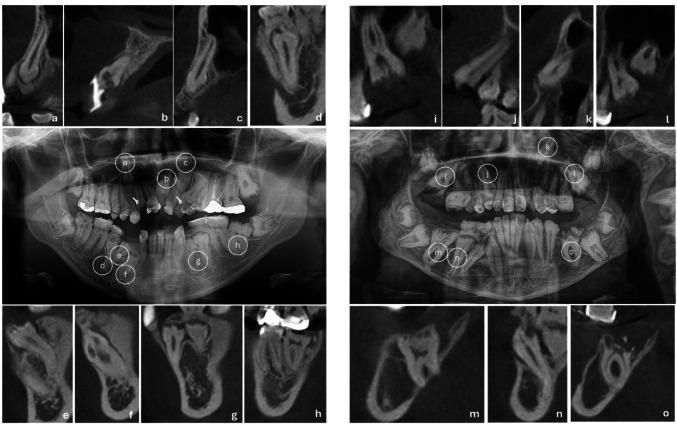
Panoramic radiographs and CBCT sagittal slices of patient 1 **a**–**h** and patient 2 **i**–**o** to show the PECR in impacted teeth. Patient 1 presented nine teeth with PECR (a to h), and patient 2 presented seven teeth with PECR **i**–**o**. In all the sagittal slices, note the irregular coronary crown surface in different degrees of severity. Some teeth presented a more severe PECR (b, f, i, k, m, and o). Enlarged dental follicles were observed in some teeth of Patient 1 **a**, **d**,**e**, **f**,**g**, and **h**) and Patient 2 **i**, **l**,**m**, **n**, and **o**. Adjacent impacted teeth presented fused dental follicles **g**, **i**, **l**, and **n**

**Fig. 3:**
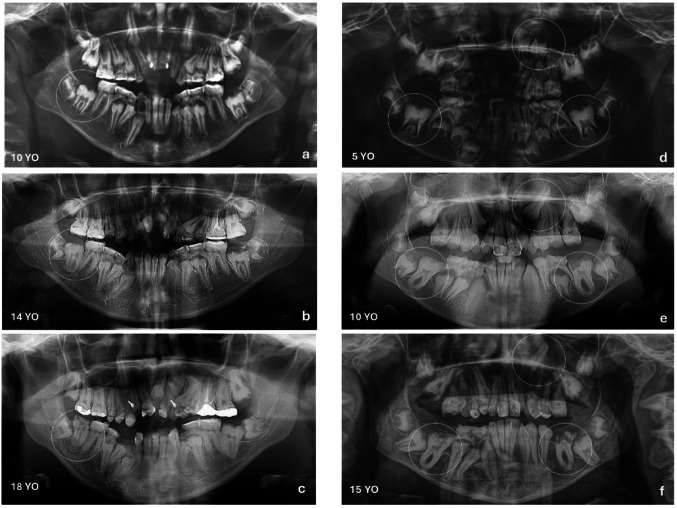
Longitudinal panoramic radiographic evaluation of Patient 1 **a**–**c** and Patient 2 **d**–**f**. **a** Panoramic radiograph of Patient 1 at age 10 shows unerupted teeth 47 (circled) without detectable resorptive changes and preserved crown morphology. **a** At age 14, PECR can be noted due to the irregular coronary surface, and at age 18, tooth 47 had already erupted, but note the extensive coronary destruction not compatible with caries decay **c** (see also the clinical aspect in Fig. 1d). **d** Panoramic radiograph of Patient 2 at age 5 reveals normal teeth development. Teeth presented normal crown morphology and surface. Teeth 23, 36, and 46 are circled. **e** At age 10, observe the unerupted teeth 23, 36, and 46 with initial signs of PECR. **f** At age 15, the progression of PECR is clearly observed

Panoramic radiographs obtained at age 18 revealed reduced enamel radiodensity, consistent with impaired enamel mineralization (Fig. 1e). All permanent teeth were present, but eleven teeth were impacted. Although the third molars remained unerupted, they were within the expected chronological sequence. Under CBCT exams, it was observed that several impacted teeth presented enlarged dental follicles (Fig. 2a, d,e, f, g, and h). Nine impacted teeth (82%) exhibited PECR (Fig. 2a–h; Table 1). Serial panoramic radiographs at ages 10, 14, and 18 showed the PECR progression in tooth 46, which exhibited a normal crown morphology at age 10, but presented a progressive PECR over time (Fig. 3a–c). PECR progression in other teeth could not be observed using panoramic radiographs.

Exome sequencing of the proband revealed a heterozygous *FAM83H* variant, causing a nonsense mutation in exon 5 (c.1055 C > A; p.Ser352*) (Table 1; Supplementary Fig. 1). The variant was predicted to be likely pathogenic (PVS1, PM2).

### Family 2

The proband was a 15-year-old Brazilian female (II:3), born to non-consanguineous parents (Fig. 1g), who was referred to the Oral Care Center for Inherited Diseases at the University Hospital of Brasília, Brazil, due to delayed tooth eruption and suspected AI. No systemic conditions were reported in the family, neither parents were affected.

Intraoral examination revealed an anterior open bite and mixed dentition (Fig. 1h–l). All teeth exhibited yellow-brown discoloration and reduced mineralization, consistent with a hypocalcified AI phenotype (Fig. 1h–j). The anterior permanent teeth in both arches had undergone previous restorative treatment, and the patient exhibited poor hygiene (Fig. 1i). Generalized gingivitis was observed. A recently erupted upper canine showed an irregular surface resembling PECR (Fig. 1l). The patient also presented with altered eruption chronology, including prolonged retention of primary teeth and delayed eruption of permanent teeth (Fig. 1k).

A panoramic radiograph at age 15 showed similar radiodensity between enamel and dentin in both primary and permanent teeth, suggesting poorly mineralized enamel. All permanent teeth were present (Fig. 1k). Eleven teeth were impacted (Fig. 1k), and seven of them were associated with PECR (63%; Fig. 2i–o; Table 1). The third molars were within the normal eruption chronology. CBCT revealed enlarged dental follicles in almost all impacted teeth (Fig. 2i, l,m, n,o). Serial panoramic radiographs obtained at 5, 10, and 15 years of age showed that teeth 26, 36, and 46 initially exhibited an apparently normal crown morphology, followed by progressive PECR over time (Fig. 3d–f).

**Fig. 4:**
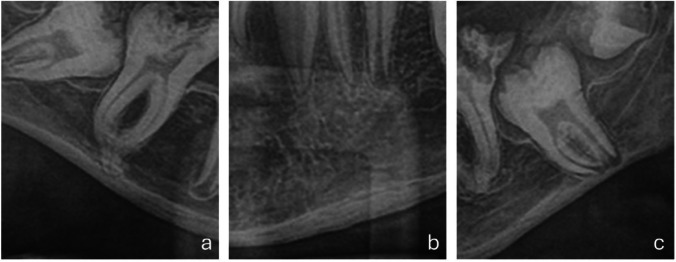
Patient 2 at age 15. Note the proximity of the root apexes of teeth 46 **a** and 36 **c** and the adjacent cortical bone. **a** The image suggests an ankylosis of the root apex and the cortical bone. **b** The basal mandibular cortex presented a localized lamellar-appearing cortical pattern

A magnified view of the lower right first molar at 15 years of age (Fig. 4a) suggested a possible fusion between the root apex and the adjacent cortical bone, raising the hypothesis of ankylosis. Tooth 36 also showed a root apex in proximity to the mandibular cortex (Fig. 4c). In addition, the patient appeared to have a narrow mandibular body. The basal mandibular cortex exhibited an unusual lamellar, layered appearance on highly magnified panoramic images (Fig. 4b). This finding was recorded descriptively and was not systematically evaluated.

Exome sequencing identified of the proband revealed a heterozygous nonsense variant in exon 5 of the *FAM83H* gene (c.1379G > A; p.Trp460*) (Table 1; Supplementary Fig. 2). This variant was predicted to be pathogenic (PS4, PVS1, PM2, PP5).

## Discussion

This study reports two unrelated Brazilian patients with ADHCAI carrying heterozygous nonsense variants in *FAM83H*, both of which correspond to previously described causative mutations. The variant identified in patient 1 (c.1055 C > A; p.Ser352*) was previously reported by Kamps et al. [18], whereas the variant c.1379G > A; p.Trp460* identified in patient 2 has been reported in five unrelated families, including the present one [3, 19–21]. The recurrence of this latter variant in multiple unrelated families supports its classification as a mutational hotspot, similar to other five recurrent variants ( c.1192 C > T, p.Gln398*; c.1289 C > T, p.Ser430*; c.1354 C > T, p.Gln452*; c.1387 C > T, p.Gln463*; c.2029 C > T, p.Gln677*) all of them located within the segment encoding Ser287–Glu694 (Supplementary S3 Table 1). All parents were clinically examined and did not present enamel defects; therefore, molecular sequencing was not performed. Considering the severe hypocalcified enamel phenotype observed in the probands, the lack of reported incomplete penetrance in *FAM83H-related* AI, and the absence of enamel defects in the parents, the findings strongly suggest an apparently *de novo* event [22].

To date, 39 causative variants in *FAM83H*, all located exclusively in exon 5, have been reported in association with ADHCAI. Among these, 92% are truncating variants, including 31 nonsense and 5 frameshift mutations. It is well established that truncating variants occurring in the last exon escape nonsense‑mediated decay, allowing the production of stable truncated proteins that exert a deleterious gain‑of‑function effect [12, 23]. Nevertheless, three heterozygous missense variants have been described in unrelated families [3, 24, 25] (Supplementary Table 1). In two of these cases, the authors reported a more attenuated hypocalcified enamel phenotype [3, 25], which aligns with the proposed disease mechanism in which stronger gain‑of‑function effects result in more severe clinical manifestations.

Beyond the characteristic hypocalcified AI and impacted teeth, our findings significantly expand the phenotypic spectrum of *FAM83H*-related ADHCAI by highlighting a high prevalence of PECR. A previous study had described PECR only in a single tooth from one patient with *FAM83H*-related AI [14] (Table 1). Here, we report PECR in 63% and 82% of the impacted teeth. In two unrelated patients and also demonstrate the clinical relevance of longitudinal radiographic monitoring and CBCT in affected individuals. An additional incidental radiographic observation in one patient was a lamellar-appearing configuration of the basal mandibular cortex on highly magnified imaging. Given that this finding was identified in a single individual and was not systematically evaluated, it should be interpreted with caution and regarded as non-specific.

Analysis of longitudinal imaging over periods ranging from 7 to 10 years showed that the affected teeth initially developed normal crown morphology, followed by the onset of PECR. This temporal progression of the coronal resorption may represent a secondary consequence of the underlying enamel defect in AI, the prolonged retention, the defective dental follicle, or all these factors. Additionally, some erupted teeth displayed crown deformities suggestive of previous resorptive activity.

The precise normal physiological function of FAM83H remains under active investigation. Expression of *FAM83H* has been documented in dental follicles, ameloblasts, and alveolar bone [15, 26]. Moreover, studies in *FAM83H* knockout mice demonstrated delayed incisor eruption, reduced pulp chamber size, and mandibular growth impairment, likely due to defective osteoblast differentiation and mineralization [9, 27]. These data suggest that *FAM83H* may play a role in the development of both epithelial and mesenchymal components of the tooth and periodontium.

The findings of the present study critically reinforce the multifaceted role of FAM83H in tooth development, extending its influence beyond enamel formation to encompass eruption and dental follicle dynamics. While impacted teeth are a recognized clinical feature in *FAM83H*-related AI [8, 15, 28], our observations specifically highlight the distinctive and severe presentation of PECR within this genetic context. PECR has been previously documented in various forms of non-syndromic and syndromic AI, linked to variants in genes such as *AMELX*, *ENAM*, *FAM20A*, and *LAMB3* [21, 29–33].

A major clinical challenge is that PECR is often misdiagnosed as mere shape anomalies or malformed teeth upon eruption. This underdiagnosis is further compounded by its asymptomatic and progressive nature, as observed in the present study and reported in patients with *LAMB3*-related AI by Besa-Witto et al. [29]. Moreover, the subtle radiographic features in early stages contribute to PECR being easily overlooked during routine assessments. In addition, subtle radiographic features in the early stages contribute to PECR being easily overlooked during routine assessments. In this study, both PECR and dental follicle enlargement were detectable only through CBCT imaging. Longitudinal evaluations, consistent with previous reports in the literature, were essential for identifying the rapid progression and severity of PECR. These analyses revealed marked advancement of resorption over relatively short periods, exemplified by the four-year interval observed in Patient 1. Furthermore, our findings demonstrate that this progressive process can ultimately lead to severe outcomes, including complete loss of the dental crown and pulp exposure, as observed in Patient 2 within a period of less than 10 years.

The mechanism of PECR remains unclear. Some hypotheses have been proposed, including failure of molecular eruption signaling from the odontogenic epithelium in patients with AI [33], immune-mediated reaction of the dental tissue, and local inflammatory reactions [34]. The presence of osteodentin-like tissue in some PECR cases [35], supports the idea of replacement resorption, a process where a resorbed dental structure is replaced by bone tissue resembling bone remodeling, suggesting an external replacement resorption [35, 36]. It has been suggested that when the protective epithelial barrier fails, multinucleated clastic cells invade the structurally compromised crown, resulting in external replacement resorption where dental tissues are substituted by trabecular bone through an active remodeling process [37–39]. The eruption process may also be affected by all these factors. In Enamel-Renal Syndrome (OMIM#204690) patients due to *FAM20A* causative variants, teeth with PECR resorption often remain unerupted, possibly due to the presence of hyperplastic dental follicles [40]. Studies suggest that the follicles of ERS patients may fail to produce eruption signals or mechanically obstruct the eruption due to cystic or fibrous transformation. Additionally, calcifications within the follicle have been reported, which could contribute to delayed eruption [41]. Similarly, in Raine syndrome (RS) (OMIM#259775), caused by FAM20C causative variants, a skeletal dysplasia, primarily associated with generalized osteosclerosis, ectopic calcifications, and hypoplastic AI, eruption disturbances involving maxillary canines and third molars are observed. These cases show no signs of DF enlargement on panoramic radiographs, although no oral CBCT data were documented [42–44]. While PECR has not been explicitly documented in RS patients, some crown deformities observed in erupted teeth, such as the semilunar‑shaped teeth described by Acevedo et al. [42], may represent clinical sequelae of undiagnosed pre‑eruptive resorptive activity, as observed in cases with *LAMB3*‑related AI [29]. The occurrence of PECR and impacted teeth in the *FAM83H* patients reported here indicates that impaction may occur in the absence of dental follicle ectopic calcifications or gingival fibromatosis. However, no histopathological analysis was performed. This observation suggests that the mechanisms leading to tooth impaction and resorption in *FAM83H* patients may differ from those described in ERS or RS syndrome. However, the overlapping pathways underlying these processes remain poorly understood, and further studies are needed to better elucidate these mechanisms and to determine how they may differ among the various forms of AI according to the specific gene involved and its role in enamel formation, dental follicle biology, and bone remodeling.

This study provides the first description of causative *FAM83H* variants in Brazilian families and reveals a previously unrecognized phenotype characterized by multiple teeth with severe pre-eruptive coronal resorption. These findings broaden the phenotypic spectrum of *FAM83H*-related ADHCAI and support the notion that causative variants in this gene may compromise dental structures beyond enamel formation. Early diagnosis and individualized radiographic monitoring are crucial to prevent extensive structural destruction and to optimize treatment planning for affected individuals.

Overall, the severity and extent of crown resorption observed here highlight the need to elucidate the mechanisms of lesion progression, including resorptive dynamics, tooth–bone interactions, and supporting bone involvement, to inform future preventive and therapeutic strategies.

**Table 1:** Reported cases of *FAM83H*-associated tooth impaction and pre-eruptive crown resorption

Author/year	Demographic origin	Sex	Age	AI phenotype	Zigosity	Variant FAM83H	Predicted protein change	Impacted tooth and pre eruptive crown resorption (PECR)
[8]	NR	M	13y	HC	HET	c.2029 C > T	p.Gln677*	
[28]	Denmark	M	12y	HC	HET	c.1354 C > T	p.Gln452*	
[45]	China	M	16y	HC	HET	c.1147G > T	p.Glu383*	
		F	14y	HC	HET	c.973 C > T	p.Arg325*	
[15]	Taiwan	F	16y	HC	HET	c.1309_1311delinsTAA	p.His437*	
		M	9y	HC	HET	c.1408 C > T	p.Gln470*	
		M	15y	HC	HET	c.1330 C > T	p.Gln444*	
		M	13y	HP	HET	c.1828G > T	p.Glu610*	
[14]	Colombia	F	29y	HC	HET	c.1289 C > A	p.Ser430*	
This study	Brazil	F	18y	HC	HET	c.1055 C > A	p.Ser352*	
		F	15y	HC	HET	c.1379G > A	p.Trp460*	

## Supplementary Information

Below is the link to the electronic supplementary material.

Supplementary Material 1

## Data Availability

The data generated and analyzed during this study can be found in the published article and its supplementary files.
