# Supplementary material for: Pre-eruptive Coronal Resorptions as a Clinical Feature of FAM83H-Related Amelogenesis Imperfecta: Insights from Two Brazilian Families
Source: Calcif Tissue Int. 2026 May 4;117(1):75. doi: 10.1007/s00223-026-01540-8 (PMC13139228; doi:10.1007/s00223-026-01540-8)
Supplement: Supplementary file 1 — Supplementary Material 1 [file 223_2026_1540_MOESM1_ESM.docx]

**Supplementary Material.**

**Pre-eruptive coronal resorptions as a clinical feature in *FAM83H*: Insights from Two Brazilian Families**

Kemelly Karolliny Moreira Resende^1,2^, Luanna de Sousa Amorim^1,2^, Lilian M Paula^1,2^, André Ferreira Leite^1,3^, Juliana Forte Mazzeu^4^, Paulo Marcio Yamaguti^1,2^ , Ana Carolina Acevedo^1,2^.

1- Department of Dentistry, Laboratory of Oral Histopathology, Faculty of Health Sciences, University of Brasilia, Brasilia, Brazil

2-Oral Care Center for Inherited Diseases, University Hospital of Brasilia, Brasilia, Brazil.

3-Department of Dentistry, Division of Radiology, Faculty of Health Sciences, University of Brasilia, Brasilia, Brazil

4-Laboratory of Clinical Genetics, Faculty of Medicine, University of Brasília, Brasília, Brazil.

**Appendix S1- Figure 1.** Integrative Genomics Viewer (IGV) visualization of the heterozygous causative variant of Patient 1 in *FAM83H*(c.1055C>A, p.Ser352Ter).


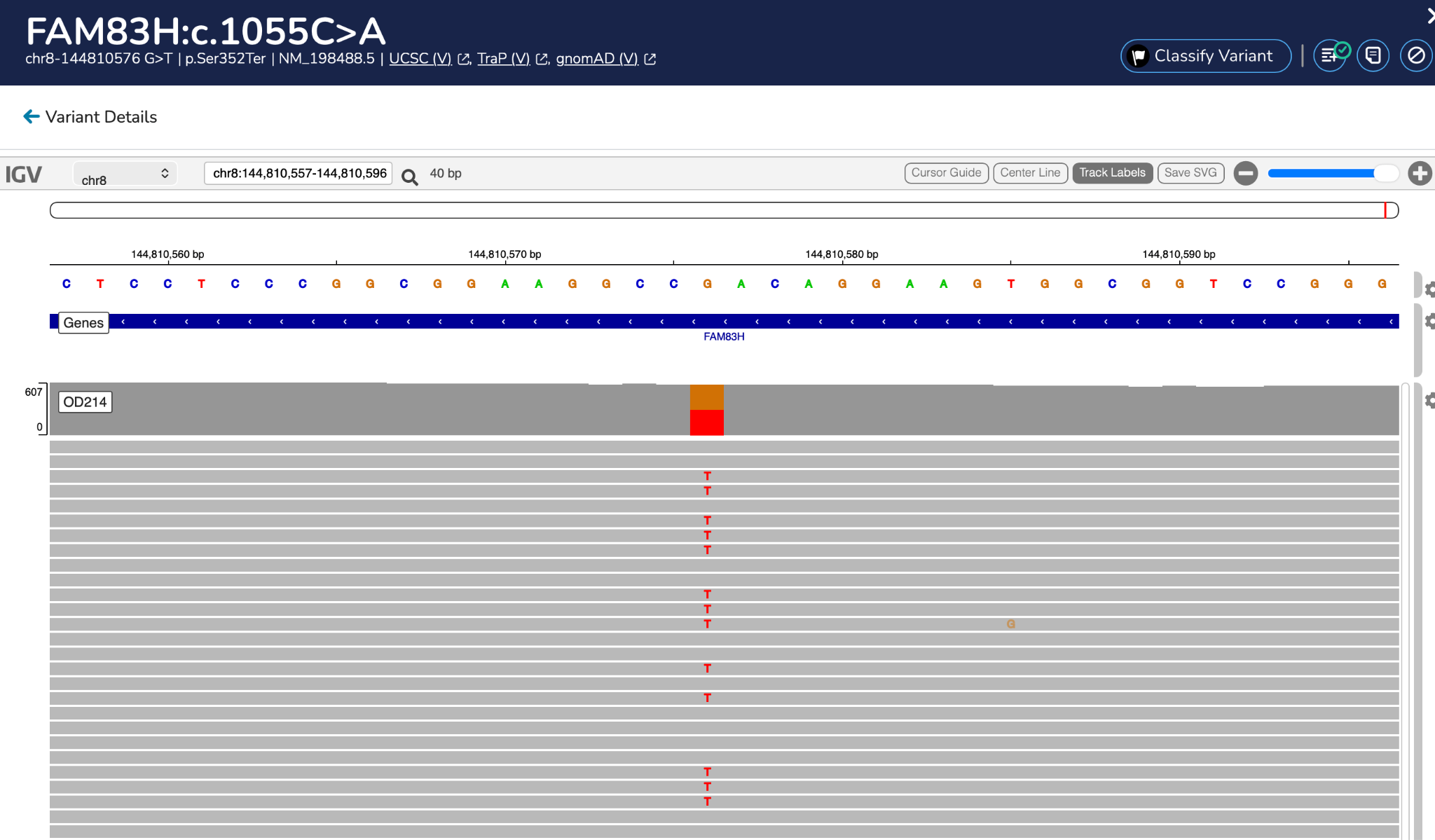


**Appendix S2 - Figure 2.** Integrative Genomics Viewer (IGV) visualization of the heterozygous causative variant of Patient 2 in *FAM83H*(c.1379G>A; p.Trp460*).


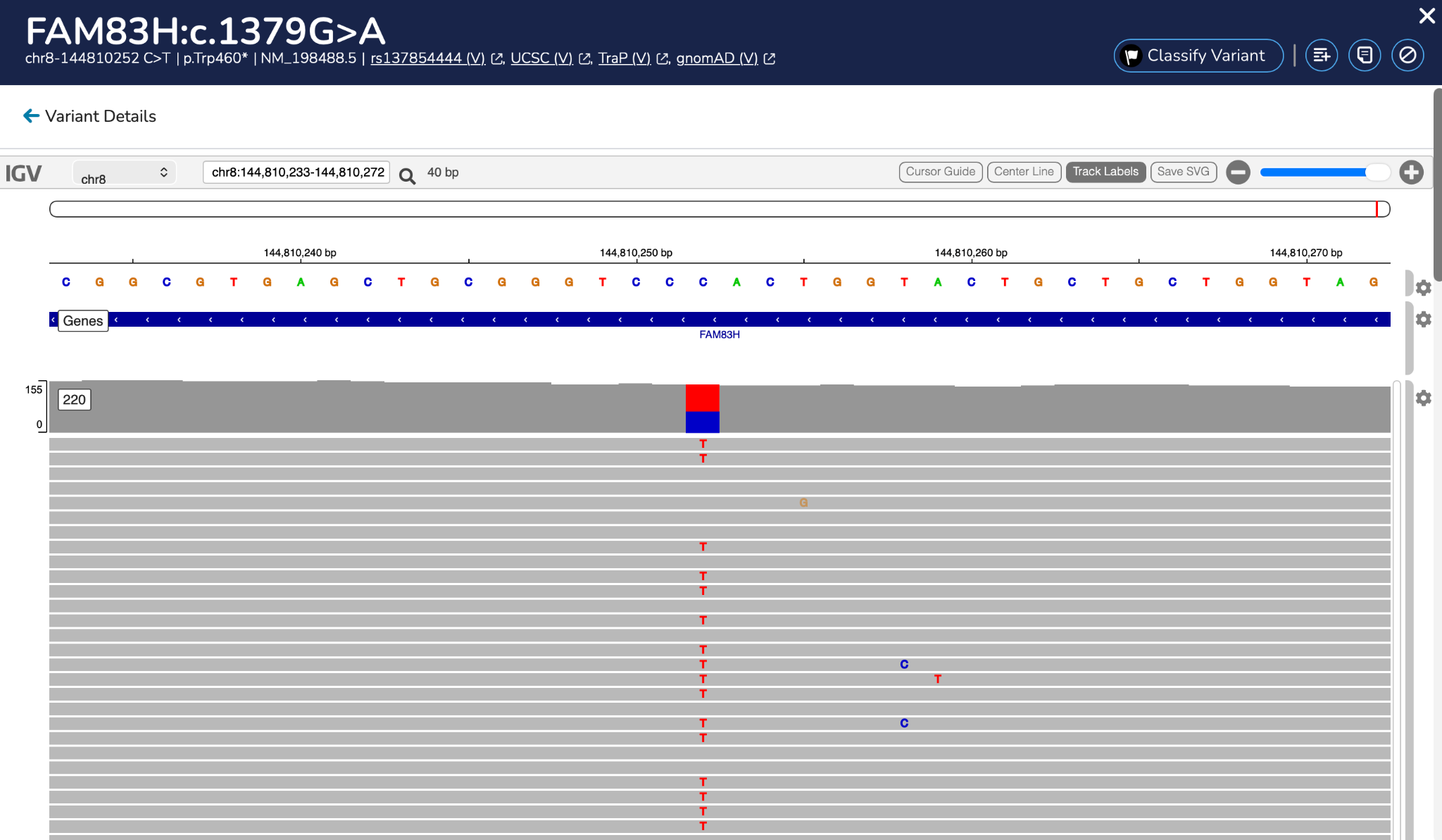


**Appendix S3-Table 1.** Description and ACMG classification of the *FAM83H* disease-causing variants reported in the literature.

| **Nº Variants** | ***FAM83H* Variants** | **Predicted protein change** | **Type of**  **Variants** | **Exon** | **ACMG**  **Classification** | **ACMG criteria** | **Reference** |
| --- | --- | --- | --- | --- | --- | --- | --- |
| 1 | c.860C>A | p.Ser287* | NS | 5 | LP | PVS1,PM2,PP5 | 1,2 |
| 2 | c.891T>A | p.Tyr297* | NS | 5 | LP | PVS1,PM2,PP5 | 3 |
| 3 | c.906T>G | p.Tyr302* | NS | 5 | LP | PVS1,PM2 | 4,5 |
| 4 | c.923_924del | p.Leu308Argfs*16 | FS | 5 | LP | PVS1,PM2,PP5 | 1,2,6 |
| 5 | c.924dup | p.Val309Argfs*16 | FS | 5 | LP | PVS1,PM2 | 3 |
| 6 | c.930_939dup | p.Val314Argfs*14 | FS | 5 | P | PS4,PVS1,PM2,PP5 | 5 |
| 7 | c.931dup | p.Val311Glyfs*14 | FS | 5 | LP | PVS1,PM2 | 7 |
| 8 | c.973C>T | p.Arg325* | NS | 5 | LP | PVS1,PM2,PP5 | 3,5-10 |
| 9 | **c.1055C>A** | **p.Ser352*** | **NS** | **5** | **LP** | **PVS1,PM2** | **11**  **this study** |
| 10 | c.1130_1131delinsAA | p.Ser377* | NS | 5 | LP | PVS1,PM2 | 7 |
| 11 | c.1147G>T | p.Glu383* | NS | 5 | LP | PVS1,PM2 | 7 |
| 12 | c.1150T>A | p.Ser342Thr | MS | 5 | NR | NR | 12 |
| 13 | c.1192C>T | p.Gln398* | NS | 5 | LP | PVS1,PM2,PP5 | 2,6,8,13-15 |
| 14 | c.1222A>T | p.Lys408* | NS | 5 | LP | PVS1,PM2 | 16 |
| 15 | c.1243G>T | p.Glu415* | NS | 5 | LP | PVS1,PM2,PP5 | 17,18 |
| 16 | c.1261G>T | p.Glu421* | NS | 5 | LP | PVS1,PM2,PP5 | 5,19 |
| 17 | c.1282C>T | p.Gln428* | NS | 5 | LP | PVS1,PM2 | 5,20 |
| 18 | c.1289C>A | p.Ser430* | NS | 5 | LP | PVS1,PM2 | 2,5,20,21 |
| 19 | c.1309_1311delinsTAA | p.His437* | NS | 5 | P | PS4,PVS1,PM2,PP5 | 6,18 |
| 20 | c.1330C>T | p.Gln444* | NS | 5 | P | PS4,PVS1,PM2,PP5 | 13,14,18 |
| 21 | c.1354C>T | p.Gln452* | NS | 5 | LP | PVS1,PM2 | 4,5,6,18,22-24 |
| 22 | c.1363C>T | p.Gln455* | NS | 5 | P | PS4,PVS1,PM2,PP5 | 3,6 |
| 23 | c.1366C>T | p.Gln456* | NS | 5 | LP | PVS1,PM2,PP5 | 14 |
| 24 | c.1369C>T | p.Gln457* | NS | 5 | LP | PVS1,PM2 | 24 |
| 25 | c.1374C>A | p.Tyr458* | NS | 5 | LP | PVS1,PM2 | 5,6,25 |
| 26 | c.1375C>T | p.Gln459* | NS | 5 | P | PS4,PVS1,PM2,PP5 | 5,18 |
| 27 | **c.1379G>A** | **p.Trp460*** | **NS** | **5** | **P** | **PS4,PVS1,PM2,PP5** | **1,2,5,26**  **this study** |
| 28 | c.1380G>A | p.Trp460* | NS | 5 | LP | PVS1,PM2,PP5 | 17 |
| 29 | c.1387C>T | p.Gln463* | NS | 5 | LP | PVS1,PM2 | 5,27,28,29 |
| 30 | c.1408C>T | p.Gln470* | NS | 5 | P | PS4,PVS1,PM2,PP5 | 1,2,18 |
| 31 | c.1498C>G | p.Leu500Val | MS | 5 | VUS | PM2,BP4 | 5 |
| 32 | c.1669G>T | p.Gly557Cys | MS | 5 | B | BS1,BS2,BP4,BP6 | 30 |
| 33 | c.1828G>T | p.Glu610* | NS | 5 | P | PS4,PVS1,PM2,PP5 | 18 |
| 34 | c.1872_1873del | p.Leu625Alafs*79 | FS | 5 | LP | PVS1,PM2,PP5 | 1,2 |
| 35 | c.1915A>T | p.Lys639* | NS | 5 | P | PS4,PVS1,PM2,PP5 | 18,24,29 |
| 36 | c.1975G>T | p.Glu659* | NS | 5 | LP | PVS1,PM2 | 31 |
| 37 | c.1993C>T | p.Gln665* | NS | 5 | LP | PVS1,PM2 | 5 |
| 38 | c.2029C>T | p.Gln677* | NS | 5 | P | PS4,PVS1,PM2,PP5 | 2,3,5,17,20,21,32 |
| 39 | c.2080G>T | p.Glu694* | NS | 5 | LP | PVS1,PM2,PP5 | 1,2 |

NS-nonsense; MS– missense; FS – frameshift; ACMG – classification of American College of Medical Genetics and Genomics; LP –likely pathogenic variant ; P – pathogenic variant; VUS–uncertain variant; NR-no report.

**REFERENCES:**

1. Wright, J. T., Frazier-Bowers, S., Simmons, D., Alexander, K., Crawford, P., Han, S. T., et al. (2009). Phenotypic variation in FAM83H-associated amelogenesis imperfecta. Journal of Dental Research, 88(4), 356–360. https://doi.org/10.1177/0022034509334217
2. Wright, J. T., Torain, M., Long, K., Seow, K., Crawford, P., Aldred, M. J., et al. (2011). Amelogenesis imperfecta: Genotype–phenotype studies in 71 families. Cells Tissues Organs, 194(2–4), 279–283. <https://doi.org/10.1159/000324342>
3. Song, Y. L., Wang, C. N., Zhang, C. Z., Yang, K., & Bian, Z. (2012). Molecular characterization of amelogenesis imperfecta in Chinese patients. Cells Tissues Organs, 196(3), 271–279. <https://doi.org/10.1159/000335607>
4. Haubek, D., Gjørup, H., Jensen, L. G., Juncker, I., Nyegaard, M., Børglum, A. D., et al. (2011). Limited phenotypic variation of hypocalcified amelogenesis imperfecta in a Danish five-generation family with a novel FAM83H nonsense mutation. International Journal of Paediatric Dentistry, 21(6), 407–412. <https://doi.org/10.1111/j.1365-263X.2011.01149.x>
5. Bloch-Zupan, A., Rey, T., Jimenez-Armijo, A., Kawczynski, M., Kharouf, N., Dure-Molla, M. de L., et al. (2023). Amelogenesis imperfecta: Next-generation sequencing sheds light on Witkop’s classification. Frontiers in Physiology, 14, 1130175. https://doi.org/10.3389/fphys.2023.1130175
6. Hany, U., Watson, C. M., Liu, L., Nikolopoulos, G., Smith, C. E. L., Poulter, J. A., et al. (2025). Genetic Screening of a Nonsyndromic Amelogenesis Imperfecta Patient Cohort Using a Custom smMIP Reagent for Selective Enrichment of Target Loci. *Human mutation*, *2025*, 8942542. <https://doi.org/10.1155/humu/8942542>
7. Xin, W., Wenjun, W., Man, Q., & Yuming, Z. (2017). Novel FAM83H mutations in patients with amelogenesis imperfecta. Scientific Reports, 7, 6075. https://doi.org/10.1038/s41598-017-06489-6
8. Kim, J. W., Lee, S. K., Lee, Z. H., Park, J. C., Lee, K. E., Lee, M. H., et al. (2008). FAM83H mutations in families with autosomal-dominant hypocalcified amelogenesis imperfecta. American Journal of Human Genetics, 82(2), 489–494. https://doi.org/10.1016/j.ajhg.2007.09.019
9. Xie, Y., Meng, M., Cao, L., Yang, J., Ma, Q., Huang, X., Yu, Y., Yang, Q., Zou, J., & Du, Q. (2023). Amelogenesis imperfecta in a Chinese family resulting from a FAM83H variation and the effect of FAM83H on the secretion of enamel matrix proteins. Clinical oral investigations, 27(3), 1289–1299. https://doi.org/10.1007/s00784-022-04763-9
10. Zhang, C., Song, Y., & Bian, Z. (2015). Ultrastructural analysis of the teeth affected by amelogenesis imperfecta resulting from FAM83H mutations and review of the literature. Oral Surgery, Oral Medicine, Oral Pathology and Oral Radiology, 119(2), e69–e76. https://doi.org/10.1016/j.oooo.2014.10.004
11. Kamps, R., Martens, H., de Koning, B., Smeets, B., & van Geel, M. (2025). Identifying a novel causal FAM83H variant for autosomal dominant amelogenesis imperfecta using exome-sequencing. Molecular Genetics & Genomic Medicine, 13(6), e70108. https://doi.org/10.1002/mgg3.701081
12. Pourhashemi, S., Ghandehari, M., Motlagh, M., Meighani, G., & Takaloo, A. (2014). Missense mutation in Fam83H gene in Iranian patients with amelogenesis imperfecta. Iranian Journal of Public Health, 43, 123–129.
13. Ding, Y., Estrella, M. R. P., Hu, Y. Y., Chan, H. L., Zhang, H. D., Kim, J. W., et al. (2009). Fam83h is associated with intracellular vesicles and ADHCAI. Journal of Dental Research, 88(11), 991–996. https://doi.org/10.1177/0022034509344564
14. Hart, P., Becerik, S., Cogulu, D., Emingil, G., Ozdemir‐Ozenen, D., Han, S., et al. (2009). Novel FAM83H mutations in Turkish families with autosomal dominant hypocalcified amelogenesis imperfecta. Clinical Genetics, 75(4), 401–404. https://doi.org/10.1111/j.1399-0004.2009.01156.x
15. Tan, L., Guo, Y., Zhong, M. M., Zhao, Y. Q., Zhao, J., Aimee, D. M., et al. (2023). Tooth ultrastructure changes induced by a nonsense mutation in the FAM83H gene: Insights into the diversity of amelogenesis imperfecta. Clinical Oral Investigations, 27(10), 6111–6123. https://doi.org/10.1007/s00784-023-05066-y
16. Yu, S., Quan, J., Wang, X., Sun, X., Zhang, X., Liu, Y., Zhang, C., & Zheng, S. (2018). A novel FAM83H mutation in one Chinese family with autosomal-dominant hypocalcification amelogenesis imperfecta. Mutagenesis, 33(4), 333–340. <https://doi.org/10.1093/mutage/gey019>
17. Lee, S. K., Hu, J. C. C., Bartlett, J. D., Lee, K. E., Lin, B. P. J., Simmer, J. P., et al. (2008). Mutational spectrum of FAM83H: The C-terminal portion is required for tooth enamel calcification. Human Mutation, 29(8), E95–E99. https://doi.org/10.1002/humu.20711
18. Wang, S. K., Zhang, H., Hu, C. Y., Liu, J. F., Chadha, S., Kim, J. W., et al. (2021). FAM83H and autosomal dominant hypocalcified amelogenesis imperfecta. Journal of Dental Research, 100(3), 293–301. https://doi.org/10.1177/0022034520977563
19. Nowwarote, N., Osathanon, T., Kanjana, K., Theerapanon, T., Porntaveetus, T., & Shotelersuk, V. (2019). Decreased osteogenic activity and mineralization of alveolar bone cells from a patient with amelogenesis imperfecta and FAM83H 1261G>T mutation. Genes & Diseases, 6(4), 391–397. https://doi.org/10.1016/j.gendis.2019.01.005
20. Prasad, M. K., Geoffroy, V., Vicaire, S., Jost, B., Dumas, M., Le Gras, S., et al. (2016). A targeted next-generation sequencing assay for the molecular diagnosis of genetic disorders with orodental involvement. Journal of Medical Genetics, 53(2), 98–110. https://doi.org/10.1136/jmedgenet-2015-103203
21. Alvarez, C., Aragón, M. A., Lee, Y., Gutiérrez, S., Méndez, P., García, D. A., et al. (2022). A recurrent FAM83H mutation in an extended Colombian family and variable craniofacial phenotypes. Children, 9(3), 362. https://doi.org/10.3390/children9030362
22. Hyun, H. K., Lee, S. K., Lee, K. E., Kang, H. Y., Kim, E. J., Choung, P. H., et al. (2009). Identification of a novel FAM83H mutation and microhardness of an affected molar in autosomal dominant hypocalcified amelogenesis imperfecta. International Endodontic Journal, 42(11), 1039–1043. https://doi.org/10.1111/j.1365-2591.2009.01608.x
23. Chan, H., Estrella, N. M. R. P., Milkovich, R. N., Kim, J., Simmer, J. P., & Hu, J. C. (2011). Target gene analyses of 39 amelogenesis imperfecta kindreds. European Journal of Oral Sciences, 119(Suppl. 1), 311–323. https://doi.org/10.1111/j.1600-0722.2011.00843.x
24. Wang, S., Hu, Y., Yang, J., Smith, C. E., Richardson, A. S., Yamakoshi, Y., et al. (2016). Fam83h null mice support a neomorphic mechanism for human ADHCAI. Molecular Genetics & Genomic Medicine, 4(1), 46–67. https://doi.org/10.1002/mgg3.187
25. El-Sayed, W., Shore, R. C., Parry, D. A., Inglehearn, C. F., & Mighell, A. J. (2010). Ultrastructural analyses of deciduous teeth affected by hypocalcified amelogenesis imperfecta from a family with a novel Y458X FAM83H nonsense mutation. Cells Tissues Organs, 191(3), 235–239. https://doi.org/10.1159/000237503
26. Wang, S. K., Hu, Y., Simmer, J. P., Seymen, F., Estrella, N. M. R. P., Pal, S., et al. (2013). Novel KLK4 and MMP20 mutations discovered by whole-exome sequencing. Journal of Dental Research, 92(3), 266–271. https://doi.org/10.1177/0022034512473111
27. Kantaputra, P. N., Intachai, W., & Auychai, P. (2016). All enamel is not created equal: Supports from a novel FAM83H mutation. American Journal of Medical Genetics Part A, 170(1), 273–276. https://doi.org/10.1002/ajmg.a.37385
28. Sriwattanapong, K., Nitayavardhana, I., Theerapanon, T., Thaweesapphithak, S., Chantarawaratit, P., Garuyakich, R., et al. (2022). Age-related dental phenotypes and tooth characteristics of FAM83H-associated hypocalcified amelogenesis imperfecta. Oral Diseases, 28(3), 734–744. <https://doi.org/10.1111/odi.13840>
29. Zheng, Y., Lu, T., Chen, J., Li, M., Xiong, J., He, F., et al. (2021). The gain-of-function FAM83H mutation caused hypocalcification amelogenesis imperfecta in a Chinese family. Clinical Oral Investigations, 25(5), 2915–2923. https://doi.org/10.1007/s00784-020-03759-1
30. Urzúa, B., Martínez, C., Ortega-Pinto, A., Adorno, D., Morales-Bozo, I., Riadi, G., et al. (2015). Novel missense mutation of the FAM83H gene causes retention of amelogenin and a mild clinical phenotype of hypocalcified enamel. Archives of Oral Biology, 60(9), 1356–1367. https://doi.org/10.1016/j.archoralbio.2015.06.007
31. Bai, R., He, W., Peng, Q., Shen, S., Yu, Q., Du, J., et al. (2022). A novel FAM83H variant causes familial amelogenesis imperfecta with incomplete penetrance. Molecular Genetics & Genomic Medicine, 10(4), e1902. https://doi.org/10.1002/mgg3.1902
32. Lee, K., Lee, S., Jung, S., Song, S. J., Cho, S. H., Lee, Z. H., et al. (2011). A novel mutation in the AMELX gene and multiple crown resorptions. European Journal of Oral Sciences, 119(Suppl. 1), 324–328. <https://doi.org/10.1111/j.1600-0722.2011.00846.x>
